# On the Role of Aminochrome in Mitochondrial Dysfunction and Endoplasmic Reticulum Stress in Parkinson's Disease

**DOI:** 10.3389/fnins.2019.00271

**Published:** 2019-03-29

**Authors:** Juan Segura-Aguilar

**Affiliations:** Molecular and Clinical Pharmacology, Faculty of Medicine, ICBM, University of Chile, Santiago, Chile

**Keywords:** mitochondrial dysfunction, dopamine, aminochrome, endoplasmic reticulum stress, Parkinson's disease, glutathione-S-transferase (GST), DT-diaphorase neurodegeneration

The identity of what triggers the loss of dopaminergic neurons containing neuromelanin in Parkinson's disease (PD) is still unknown. Fifty years since its introduction in PD therapy, L-dopa is still the gold-standard drug despite severe side effects observed after 4 to 6 years of being treated with it. There are no new therapies that can halt or slow down the progression of the disease and much of the research efforts in this context have been destined to treat L-dopa-induced dyskinesia. There is huge concern about the difficulties that have been observed in the translation of successful preclinical results into clinical studies and new therapies in PD. The discovery of genes associated with familiar forms of PD has made an enormous input into basic research, which seeks to understand the degenerative process resulting in the loss of dopaminergic neurons in the nigrostriatal system. Several mechanisms have been suggested to be involved in the degeneration of nigrostriatal neurons in PD, including mitochondrial dysfunction, endoplasmic reticulum stress, lysosomal and proteasomal protein degradation dysfunction, the formation of neurotoxic alpha-synuclein (SNCA) oligomers, neuroinflammation, and oxidative stress.

## Mitochondrial Dysfunction

The brain is completely dependent on chemical energy (ATP) in order to perform the release of neurotransmitters such as dopamine. Therefore, the existence of functional mitochondria is essential to the performed role of a dopaminergic neuron, i.e., to release dopamine. Postmortem brains with PD presented a deficiency in Complex I activity (Shapira et al., 1990; Esteves et al., 2011). Reduced Complex I activity in platelet mitochondria, purified from patients with idiopathic PD, has been observed (Esteves et al., 2011). CHCHD2 mutation in PD patient fibroblasts reduces oxidative phosphorylation in Complexes I and IV and induces fragmentation of the mitochondrial reticular morphology (Lee et al., 2018). A meta-analysis supports the deficit in Complexes I and IV in the case of peripheral blood, the frontal cortex, the cerebellum and the substantia nigra in PD (Holper et al., 2018). Analysis of mitochondria morphology in PD samples compared to controls revealed a significant decrease in the number of healthy mitochondria per cell. Several genes associated with familial forms of PD (PINK-1, DJ-1, Parkin, HTRA2) are linked to mitochondrial impairment (Larsen et al., 2018). Parkinson's disease, associated with vacuolar protein sorting 35 mutation, affects Complex I activity (Zhou et al., 2017). PINK1 and DJ-1 mutation induce energetic inefficiency (Lopez-Fabuel et al., 2017). SNCA induces mitochondrial dysfunction (Devi et al., 2008; Chinta et al., 2010; Nakamura et al., 2011; Martínez et al., 2018).

## Endoplasmic Reticulum Stress

Endoplasmic reticulum is involved in secretory protein translocation and the quality control of secretory protein folding. Misfolded or unfolded proteins in the lumen accumulate under endoplasmic reticulum stress, which causing an integrated adaptive response identified as the unfolded protein response (UPR), which seeks to restore proteostasis within the secretory pathway (Cabral-Miranda and Hetz, 2018).

The UPR activation markers, phosphorylated eukaryotic initiation factor 2alpha and phosphorylated pancreatic endoplasmic reticulum kinase, were detected in dopaminergic neurons containing neuromelanin in the substantia nigra of PD patients. Interestingly, phosphorylated pancreatic endoplasmic reticulum kinase was colocalized with an increased level of SNCA (Hoozemans et al., 2007). Neuropathological analysis of PD postmortem brain tissue revealed that pIRE1α is expressed within neurons containing elevated levels of α-synuclein or Lewy bodies (Heman-Ackah et al., 2017). SNCA triggers endoplasmic reticulum stress via the protein kinase RNA-like endoplasmic reticulum kinase/eukaryotic translation initiation factor 2α signaling pathway (Liu et al., 2018). N370S mutation and β-glucocerebrosidase-1 retention within the endoplasmic reticulum induce endoplasmic reticulum stress activation, triggering UPR and Golgi apparatus fragmentation (García-Sanz et al., 2017). It has been reported that endoplasmic reticulum stress activates the chaperone-mediated autophagy pathway via an EIF2AK3/PERK-MAP2K4/MKK4-MAPK14/p38-dependent manner (Li et al., 2018).

## Dopamine Oxidation and Parkinson's Disease

One of the most characteristic features of the pathology of PD, which results in the onset of motor symptoms, is the massive loss of dopaminergic neurons containing neuromelanin in the nigrostriatal system. As mentioned before, several mechanisms, including mitochondrial dysfunction and endoplasmic reticulum stress, have been proposed as being involved in the degeneration of the nigrostriatal neurons in PD, but the question concerns what triggers these mechanisms in dopaminergic neurons containing neuromelanin. Many times, it has been suggested that the involvement of exogenous neurotoxins triggers these mechanisms, but the severe Parkinsonism induced by MPTP in just 3 days in drug addicts who used synthetic drugs contaminated with this compound undermines this idea (Williams, 1986). The rate of the degenerative process in PD takes years (Braak et al., 2004). The extremely slow degeneration of the nigrostriatal neurons and slow progression of the disease challenge the possible role of exogenous neurotoxins in the loss of dopaminergic neurons containing neuromelanin, suggesting that some endogenous neurotoxin must trigger these mechanisms. A neurotoxic event, triggered by an endogenous neurotoxin, will affect a single neuron without propagative effects, which explains the extremely slow rate of this degenerative process in PD. Among possible endogenous neurotoxins are the neurotoxic SNCA oligomers. However, the prion-like hypothesis of SNCA in PD pathogenesis is based on the propagation (neuron-to-neuron transfer) of neurotoxic SNCA oligomers (Brundin and Melki, 2017). According to this prion-like hypothesis, a relatively rapid process is expected, in contrasting with what happens in PD, which takes years. In addition, what triggers the formation of neurotoxic SNCA oligomers inside the dopaminergic neurons containing neuromelanin? Braak stage hypothesis use the intraneuronal inclusion bodies to follow the development of Parkinson's disease where SNCA is one of the aggregated proteins (Braak et al., 2004). What induces SNCA aggregation in other brain region involved in non-motor symptoms remains unclear. A possible explanation is that an endogenous neurotoxin is formed inside dopaminergic neurons containing neuromelanin during dopamine oxidation. The formation of the pigment called neuromelanin in these neurons is the result of dopamine oxidation into ortho(*o*)-quinones, which is a pathway that involves the formation of three *o*-quinones in a sequential manner (dopamine → dopamine *o*-quinone → aminochrome → 5,6- indolequinone → neuromelanin).

Dopamine *o*-quinone is able to form adducts with proteins, such as ubiquitin carboxy-terminal hydrolase L1 (UCHL-1) and Parkinsonism-associated deglycase (DJ-1, PARK7), as well as ubiquinol-cytochrome c reductase core protein 1, glucose-regulated protein 75/mitochondrial HSP70/mortalin, mitofilin, mitochondrial creatine kinase and glutathione peroxidase-4, and a human dopamine transporter (Whitehead et al., 2001; Van Laar et al., 2009; Hauser et al., 2013). Incubation of purified tyrosine hydroxylase with dopamine and tyrosinase also forms adducts with dopamine (Xu et al., 1998). Dopamine *o*-quinone induces mitochondrial dysfunction (Berman and Hastings, 1999). Exposure of cells to dopamine induced the formation of dopamine adducts with parkin (LaVoie et al., 2005), but the identity of the *o*-quinone involved in this reaction (dopamine *o*-quinone or aminochrome) is not clear. Dopamine *o*-quinone is completely unstable at physiological pH and cyclizes immediately into aminochrome; thus, the question concerns whether dopamine *o*-quinone has the opportunity to form adducts with parkin in the cell cytosol overcrowded with other proteins, molecules and organelles.

**Figure 1:**
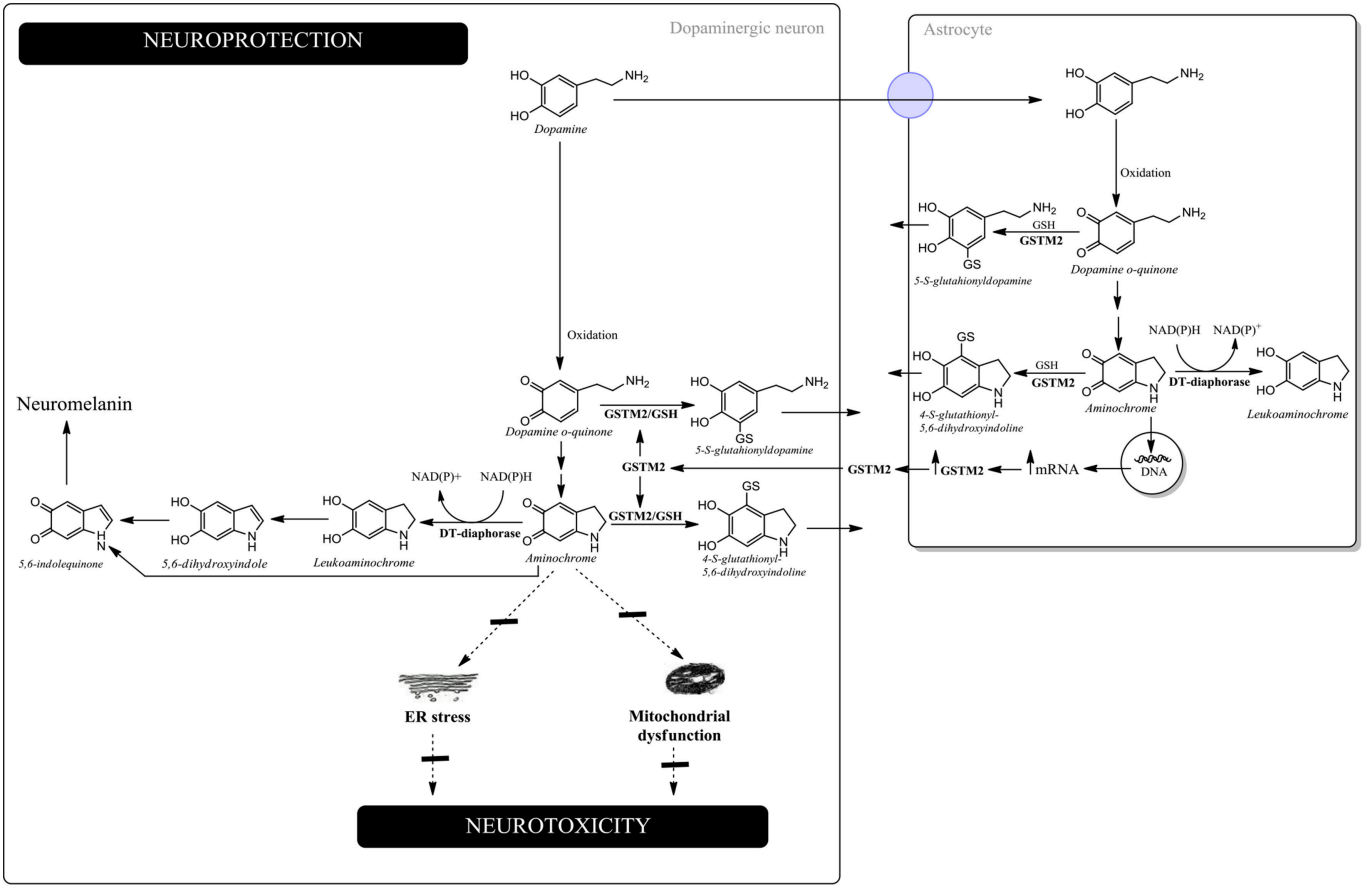
Neuroprotection against aminochrome-induced neurotoxicity. In dopaminergic neurons, DT-diaphorase catalyzes the two-electron reduction of aminochrome into leukoaminochrome, preventing aminochrome-induced endoplasmic reticulum stress and mitochondrial dysfunction. Leukoaminochrome is rearranged into 5,6-dihydroxyindole, which oxidizes into 5,6-indolequinone and polymerizes into neuromelanin. In astrocytes, GSTM2 is able to conjugate both dopamine *o*-quinone and aminochrome with GSH and DT-diaphorase can reduce aminochrome with two-electron to leukoaminochrome. However, astrocytes secrete the enzyme GSTM2, whose dopaminergic neurons internalize in the cytosol. GSTM2 inside the dopaminergic neurons conjugates both dopamine *o*-quinone and aminochrome with GSH, whose stable products are eliminated from dopaminergic neurons.

Aminochrome has been reported to be neurotoxic on account of inducing mitochondrial dysfunction, endoplasmic reticulum stress, autophagy dysfunction, proteasomal dysfunction, oxidative stress, neuroinflammation, the disruption of the cytoskeleton architecture and the formation of neurotoxic SNCA oligomers (Arriagada et al., 2004; Zafar et al., 2006; Fuentes et al., 2007; Zhou and Lim, 2009; Paris et al., 2010, 2011; Aguirre et al., 2012; Muñoz et al., 2012, 2015; Huenchuguala et al., 2014, 2017; Xiong et al., 2014; Briceño et al., 2016; Santos et al., 2017; de Araújo et al., 2018; Segura-Aguilar and Huenchuguala, 2018) (Figure 1).

5,6-Indolequinone, the precursor of neuromelanin, is able to form adducts with SNCA (Bisaglia et al., 2010). Dopaminochrome has also been reported to form adducts with SNCA (Norris et al., 2005) and to be neurotoxic in cell cultures (Linsenbardt et al., 2009, 2012). The unilateral injection of dopaminochrome induced degeneration of the dopaminergic neurons within the substantia nigra (Touchette et al., 2015). However, the structure of dopaminochrome has not been determined by NMR; nor do we know the nature of this structure. The dopaminochrome structure is different to the aminochrome structure because dopaminochrome has an absorption maximum of 303 and 479 nm (Ochs et al., 2005), while aminochrome has an absorption maximum of 280 and 475 nm and its structure has been confirmed by NMR (Paris et al., 2010).

## Aminochrome and Parkinson's Disease

Dopamine oxidation into neuromelanin is a normal and harmless pathway because neuromelanin accumulates with age, with dopaminergic neurons containing neuromelanin remaining intact in the substantia nigra of healthy seniors (Zecca et al., 2002). Aminochrome is the most stable and studied *o*-quinone formed during dopamine oxidation into neuromelanin. Paradoxically, aminochrome under certain conditions can be neurotoxic as a result of inducing mitochondrial dysfunction (Arriagada et al., 2004; Paris et al., 2011; Aguirre et al., 2012; Huenchuguala et al., 2017; Segura-Aguilar and Huenchuguala, 2018), endoplasmic reticulum stress (Xiong et al., 2014), the formation of neurotoxic SNCA oligomers (Muñoz et al., 2015; Muñoz and Segura-Aguilar, 2017), proteasome dysfunction (Zafar et al., 2006; Zhou and Lim, 2009), autophagy dysfunction (Muñoz et al., 2012; Huenchuguala et al., 2014), lysosome dysfunction (Meléndez et al., 2018), neuroinflammation (Santos et al., 2017; de Araújo et al., 2018), cytoskeleton architecture disruption (Paris et al., 2010; Briceño et al., 2016) and oxidative stress (Arriagada et al., 2004). Aminochrome *in vivo* induces neuronal dysfunction as a consequence of mitochondrial dysfunction, decreased axonal transport resulting in a significant decrease in the number of synaptic monoaminergic vesicles, reduced dopamine release accompanied by an increase in GABA levels, and a dramatic change in the neurons' morphology characterized as cell shrinkage (Herrera et al., 2016). The explanation as to why dopamine oxidation into neuromelanin is not a harmful pathway, despite the formation of potential neurotoxic *o*-quinones, is because the existence of two enzymes [DT-diaphorase and glutathione transferase M2-2 (GSTM2)], which are able to prevent aminochrome neurotoxicity. DT-diaphorase is expressed in dopaminergic neurons and astrocytes and catalyzes the two-electron reduction of aminochrome into leukoaminochrome, preventing aminochrome one-electron reduction into the leukoaminochrome *o*-semiquinone radical, catalyzed by flavoenzymes that transfer one electron and use NADH or NADPH. DT-diaphorase prevents aminochrome-induced cell death (Lozano et al., 2010), mitochondrial dysfunction (Arriagada et al., 2004; Paris et al., 2011; Muñoz et al., 2012), cytoskeleton architecture disruption (Paris et al., 2010), lysosomal dysfunction (Meléndez et al., 2018), the formation of neurotoxic SNCA oligomers (Muñoz et al., 2015; Muñoz and Segura-Aguilar, 2017), oxidative stress (Arriagada et al., 2004); dopaminergic neurons' degeneration *in vivo* (Herrera-Soto et al., 2017) and astrocytes dell death (Huenchuguala et al., 2016). GSTM2 catalyzes the GSH conjugation of aminochrome into 4-S-glutathionyl-5,6-dihydroxyindoline, which is resistant to biological oxidizing agents such as oxygen, hydrogen peroxide, and superoxide (Segura-Aguilar et al., 1997). GSTM2 also catalyzes the GSH conjugation of dopamine *o*-quinone into 5-glutathionyl-dopamine (Dagnino-Subiabre et al., 2000), which degrades into 5-cysteinyl-dopamine. Interestingly, 5-cysteinyl-dopamine is a stable metabolite that can be eliminated from the cells. 5-Cysteinyl-dopamine has been found in substantia nigra, caudate nucleus, putamen, globus pallidus, neuromelanin, and the cerebrospinal fluid of PD patients (Rosengren et al., 1985; Carstam et al., 1991; Cheng et al., 1996). GSTM2 prevents aminochrome-induced cell death, mitochondrial dysfunction, autophagy, and lysosome dysfunction (Huenchuguala et al., 2014; Segura-Aguilar, 2017a; Segura-Aguilar and Huenchuguala, 2018). The GSH conjugation of aminochrome prevents the formation of neurotoxic SNCA oligomers by generating nontoxic SNCA oligomers (Huenchuguala et al., 2018). GSTM2 is expressed in human astrocytes and it has been reported that astrocytes secrete GSTM2, while dopaminergic neurons are able to internalize this enzyme into the cytosol, protecting these neurons against aminochrome-induced neurotoxicity (Cuevas et al., 2015; Segura-Aguilar, 2015, 2017b).

Mitochondrial dysfunction and endoplasmic reticulum stress are two very important mechanisms involved in the loss of dopaminergic neurons containing neuromelanin in the nigrostriatal neurons in idiopathic PD. However, the question concerns the common denominator in these mechanisms: i.e., what triggers these mechanisms in dopaminergic neurons containing neuromelanin in the nigrostriatal system? We propose that aminochrome is the endogenous neurotoxin that triggers mitochondrial dysfunction and endoplasmic reticulum stress because aminochrome is formed inside dopaminergic neurons of the nigrostriatal system. In addition, aminochrome also triggers other mechanisms involved in the loss of dopaminergic neurons in the nigrostriatal system, such as the formation of neurotoxic SNCA oligomers, oxidative stress, neuroinflammation, and proteasomal and lysosomal protein degradation dysfunction.

## Author Contributions

The author confirms being the sole contributor of this work and has approved it for publication.

### Conflict of Interest Statement

The author declares that the research was conducted in the absence of any commercial or financial relationships that could be construed as a potential conflict of interest.
